# Orexin-A Promotes Cell Migration in Cultured Rat Astrocytes via Ca^2+^-Dependent PKCα and ERK1/2 Signals

**DOI:** 10.1371/journal.pone.0095259

**Published:** 2014-04-18

**Authors:** Qing Shu, Zhuang-Li Hu, Chao Huang, Xiao-Wei Yu, Hua Fan, Jing-Wen Yang, Peng Fang, Lan Ni, Jian-Guo Chen, Fang Wang

**Affiliations:** 1 Department of Pharmacology, Tongji Medical College, Huazhong University of Science and Technology, Wuhan, Hubei, China; 2 Key Laboratory of Neurological Diseases (HUST), Ministry of Education of China, Wuhan, Hubei, China; 3 The Key Laboratory for Drug Target Researches and Pharmacodynamic Evaluation of Hubei Province, Wuhan, Hubei, China; University of Louisville, United States of America

## Abstract

Orexin-A is an important neuropeptide involved in the regulation of feeding, arousal, energy consuming, and reward seeking in the body. The effects of orexin-A have widely studied in neurons but not in astrocytes. Here, we report that OX1R and OX2R are expressed in cultured rat astrocytes. Orexin-A stimulated the phosphorylation of extracellular signal-regulated kinase 1/2 (ERK1/2), and then induced the migration of astrocytes via its receptor OX1R but not OX2R. Orexin-A-induced ERK1/2 phosphorylation and astrocytes migration are Ca^2+^-dependent, since they could be inhibited by either chelating the extracellular Ca^2+^ or blocking the pathway of store-operated calcium entry (SOCE). Furthermore, both non-selective protein kinase C (PKC) inhibitor and PKCα selective inhibitor, but not PKCδ inhibitor, prevented the increase in ERK1/2 phosphorylation and the migration of astrocytes, indicating that the Ca^2+^-dependent PKCα acts as the downstream of the OX1R activation and mediates the orexin-A-induced increase in ERK1/2 phosphorylation and cell migration. In conclusion, these results suggest that orexin-A can stimulate ERK1/2 phosphorylation and then facilitate the migration of astrocytes via PLC-PKCα signal pathway, providing new knowledge about the functions of the OX1R in astrocytes.

## Introduction

Orexins, also known as hypocretins, are a pair of neuropeptides that first discovered in a specific population of neurons in the lateral hypothalamic area (LHA) [1], [2], a region of the brain implicated in feeding, arousal, and motivated behavior. Orexin-A (hypocretin-1) and orexin-B (hypocretin-2) are derivatives from a common precursor, prepro-orexin [3]. They exert their actions via interaction with two closely related GPCRs called orexin type 1 receptor (OX1R) and 2 (OX2R) [4]. OX1R couples exclusively to pertussis toxin-insensitive G proteins, while OX2R couples to both pertussis toxin-insensitive and phospholipase C (PLC)-sensitive G proteins [5]. OX1R has greater affinity to orexin-A than orexin-B by 1 order of magnitude. In contrast, OX2R has similar affinity for both orexin-A and orexin-B [5]. The bulk of evidence, obtained in a Chinese hamster ovary (CHO) cell line stably expressing OX1R and/or OX2R, indicates that activation of both receptors increases intracellular Ca^2+^ concentration [6], [7]. However, little is known about the intracellular events set off by orexins in astrocytes.

Astrocytes, the largest population of non-excitable cells in the central nervous system (CNS), are initially considered as supporting cells in CNS. However, they are now regarded as a syncytium of interconnected cells, rather than as individual bodies to maintain normal neurological functions. In most conditions, the effects of astrocytes are mainly mediated by their membrane receptors such as AMPA receptors [8]. A previous study reported that activation of OX1R stimulates cAMP synthesis in primary rat astrocytes [9]. However, they did not certify whether OX1R exists in astrocytes, and the role of OX1R activation in astrocytes is still obscure. Considering the complexity of orexin signaling transduction pathways, we asked whether other signal molecules could mediate the effects of orexin-A on astrocytes.

Extracellular signal-regulated kinase 1/2 (ERK1/2) is a potential candidate for this supposition since ERK1/2 is reported to mediate orexin functions in several cell types [10], [11]. As we known, ERK1/2 is a member of mitogen-activated protein kinase (MAPK) family, whose activation in response to stimuli is involved in cell migration. For instance, ERK1/2 activation can mediate bradykinin-induced astrocyte migration [12]. Migration is a fundamental property of cells that occurs during many physiological and pathological processes including organogenesis in the embryo, tissue repair following injury, the inflammatory response, the formation of new blood vessels, and the spread of cancer. OX1R activation mediates MAPK cascades in numerous cells, including endothelial cells [13] and human H295R adrenocortical cells [14]. However, it is still unclear whether OX1R can mediate cell migration via ERK1/2 pathway in cultured astrocytes.

In the present study, our results demonstrated the expression of OX1R and OX2R in the rat astrocytes. It was also shown that orexin-A promoted astrocytes migration by activation of ERK1/2 via augmenting OX1R-PLC-protein kinase Cα (PKCα) signals in cultured astrocytes. In addition, intracellular Ca^2+^ contributed to orexin-A-induced ERK1/2 phosphorylation and astrocyte migration. In general, our results might provide new perspectives to understand the roles of orexin in central nervous system.

## Materials and Methods

### Materials

BAPTA-AM, U73122, 2-aminoethoxy-diphenylborate (2-APB), cyclopiazonic acid (CPA), GF109203X and poly-L-lysine were purchased from Sigma-Aldrich (St. Louis, MO, USA). Orexin-A, SB334867, Gö6976, Rottlerin and TCS OX2 29 were purchased from Tocris (Bristol, UK). U0126 was purchased from cell signaling (Boston, MA, USA). Fura-2/AM and Dulbecco's modified Eagle's medium DMEM/F12 were obtained from Biotium (San Francisco, CA, USA) and Gibco Invitrogen Corporation (Carlsbad, CA, USA), respectively. Anti-ERK and anti-p-ERK antibodies were purchased from Cell Signaling (Boston, MA, USA); Anti-β-actin, anti-OX1R and anti-OX2R antibodies were purchased from Santa Cruz (Dallas, Texas, USA). Anti-GFAP was purchased from Abcam (Cambridge, MA, USA). Both goat anti-mouse and goat anti-rabbit HRP IgG polyclonal secondary antibodies were purchased from Thermo Scientific (Rockford, IL, USA). Other general agents were purchased from commercial suppliers.

All the drugs were prepared as stock solutions. Orexin-A, TCS OX2 29 and poly-L-lysine were dissolved in the distilled water. Fura-2/AM, U73122, SB334867, 2-APB, U0126, GF109203X, Rottlerin, Gö6976, BAPTA-AM, and CPA were dissolved in dimethylsulfoxide (DMSO). All stock solutions were stored at −20°C. These stock solutions were diluted to the final concentrations with the extracellular solution before application. The final concentration of DMSO was <0.05%. No detectable effect of DMSO was found in the experiments.

### Isolation and culture of primary rat astrocyte

Neonatal Sprague-Dawley (SD) rats (day 0–3) were obtained from the Experimental Animal Center of Tongji Medical College, Huazhong University of Science & Technology. All animal care and experimental procedures complied with local and international guidelines on ethical use of animals and were approved by The University Animal Welfare Committee, Tongji Medical College, Huazhong University of Science & Technology. To obtain astrocyte, cortical tissues were isolated as previously described with some modifications [15]. Briefly, postnatal day 1–3 SD rats were decapitated, and the cortices were removed and digested with 0.125% trypsin for 20 min at 37°C. After trituration and centrifugation at 118 g for 6 min, the cells were resuspended and plated on culture flasks coated with 1 mg/mL poly-L-lysine. The cells were then cultured in DMEM and F12 (1∶1) supplemented with 10% fetal bovine serum (heat-inactivated; HyClone, Logan, UT), 2 mM L-glutamine, and 100 U/mL penicillin-streptomycin. After 24 h, the medium was changed to fresh DMEM/F12 and replaced every 3–4 days. These mixed glial cells were cultured for 9 days at 37°C in a humidified 5% CO_2_ atmosphere incubator. Astrocytes were purified from the mixed culture by mild shaking (37°C, 150 g/min, 15 h), as described previously [15]. The isolated astrocytes were cultured in DMEM/F12.

### Western blotting

After boiling in SDS sample buffer (5 min), equal amounts of protein were loaded in each lane and separated on 10% SDS/PAGE gels. Samples containing 20 µg proteins were separated electrophoretically and then transferred to nitrocellulose membranes by using a transfer cell system (Bio-Rad, California, USA). After blocking with 5% nonfat dried milk powder/TBS/0.1% Tween 20 (1 h at room temperature), membranes were probed with the appropriate antibodies overnight at 4°C. Membrane-bound primary antibodies were detected using secondary antibodies conjugated with horseradish peroxidase (1∶10000). Immunoblots were developed on Microchemi (DNR, Jerusalem, Israel) using the enhanced chemiluminescence technique (ECL; Pierce, Rockford, USA). All assays were performed at least three times.

### Calcium imaging

Primary astrocyte grown on glass cover slips were washed three times with extracellular solution containing (mM): NaCl 140, KCl 5, MgCl_2_ 1, CaCl_2_ 2, Glucose 10 and HEPES 10 (pH 7.4) and incubated with 1.5 µg/ml Fura-2/AM for 30 min at 37°C. CaCl_2_ was omitted and 0.1 mM EGTA was added in Ca^2+^–free solutions. After additional washes, coverslips with Fura-2/AM-loaded cells were mounted on a chamber positioned on the movable stage of an inverted microscope (Nikon, Tokyo, Japan) illuminated at 340 and 380 nm using calcium imaging system (PTI, Birmingham, NJ, USA). Paired F340/F380 fluorescence ratio images were acquired every second for [Ca^2+^]_i_ and the emitted light was imaged at 510 nm with a video camera (PTI Image) through a X-70 fluor oil immersion lens (Nikon, Tokyo, Japan) and a 460 nm long-pass barrier filter. Ratio images (340 nm/380 nm) were analyzed by Metafluor software (LLC, Sunnyvale, CA, USA). The values were exported to Sigmaplot 10.0 for further analysis, and the [Ca^2+^]_i_ response amplitude was normalized to the basal amplitudes, which was taken as 1.

### Cell migration (wound healing) assay

Astrocyte cells were grown to confluence in dishes and starved with serum-free DMED/F12 medium for 24 h. The monolayer cells were manually scratched in the center of the dishes with a bright and clear field. The detached cells were removed by washing the cells once with PBS. Serum-free DMEM/F12 medium with or without orexin-A was added to each dish as indicated after pretreatment with the inhibitor for 30 min. Then the cells were separately fixed with 4% paraformaldehyde and labeled with anti-GFAP (1∶200) for photography and analyzing of the cell migration at 0, 24 and 48 h. Images of migratory cells from the scratched boundary were observed and acquired at different time with a digital camera and a light microscope (Olympus, Japan). The images were exported to Sigmaplot 10.0 for further analysis, the boundary of cell migration was defined by average displacement from the dotted line, and the migration distance and migration area were normalized to the control level, which was taken as 1. The figure represents one of four individual experiments.

### Immunofluorescence assay of astrocytes *in vitro*

Cultured astrocytes were fixed with 4% paraformaldehyde in 0.01 M phosphate-buffered saline (PBS, pH 7.4) for 30 min and then rinsed three times with PBS for 10 min each. After that, they were then permeabilized in PBS with 0.3% Triton X-100 for 30 min, followed by 3% BSA-PBS for 30 min at room temperature. The cells were co-incubated with 1∶50 anti-OX1R, anti-OX2R, and anti-GFAP antibody in PBS/0.3% Triton X-100/1% BSA/2% goat serum overnight at 4°C. Cells were rinsed in PBS three times for 10 min each, and then co-incubated with different second antibody in PBS containing 0.3% TritonX-100, 2% goat serum and 1% BSA for 1 h at room temperature. After washed three times with PBS, cells were mounted on glass slides with 10%-15% glycerin and imaged using a confocal laser scanning microscope (FV500; Olympus, Tokyo, Japan).

### RNA interference

A third generation of Self-inactivating lentivirus vector (GeneChem, Shanghai, China) containing a CMV-driven GFP reporter and a U6 promoter upstream of the cloning sites (Age I and EcoR I) was used for cloning small hairpin RNAs (shRNAs). The target sequence for rat orexin receptor 1 (OX1R) was 5′-CAGATGAACTCTACCCTAA-3′; the negative control sequence was 5′-TTCTCCGAACGTGTCACGT-3′. The astrocyte was infected with lentivirus at a multiplicity of infection (MOI) of 20 for 8 h. Then the medium was replaced with fresh complete medium. After 3 days, cells were observed under fluorescence microscopy to confirm that more than 80% of cells were GFP-positive.

### Data analysis

Data were expressed as mean ± SEM. Student's *t* test and ANOVA were used for statistical analysis where appropriate. The software SPSS 13.0 and Sigmaplot 10.0 were used. The criteria of significance were set at p<0.05.

## Results

### Orexin-A promotes cell migration in primary cultured rat astrocytes

**Figure 1:**
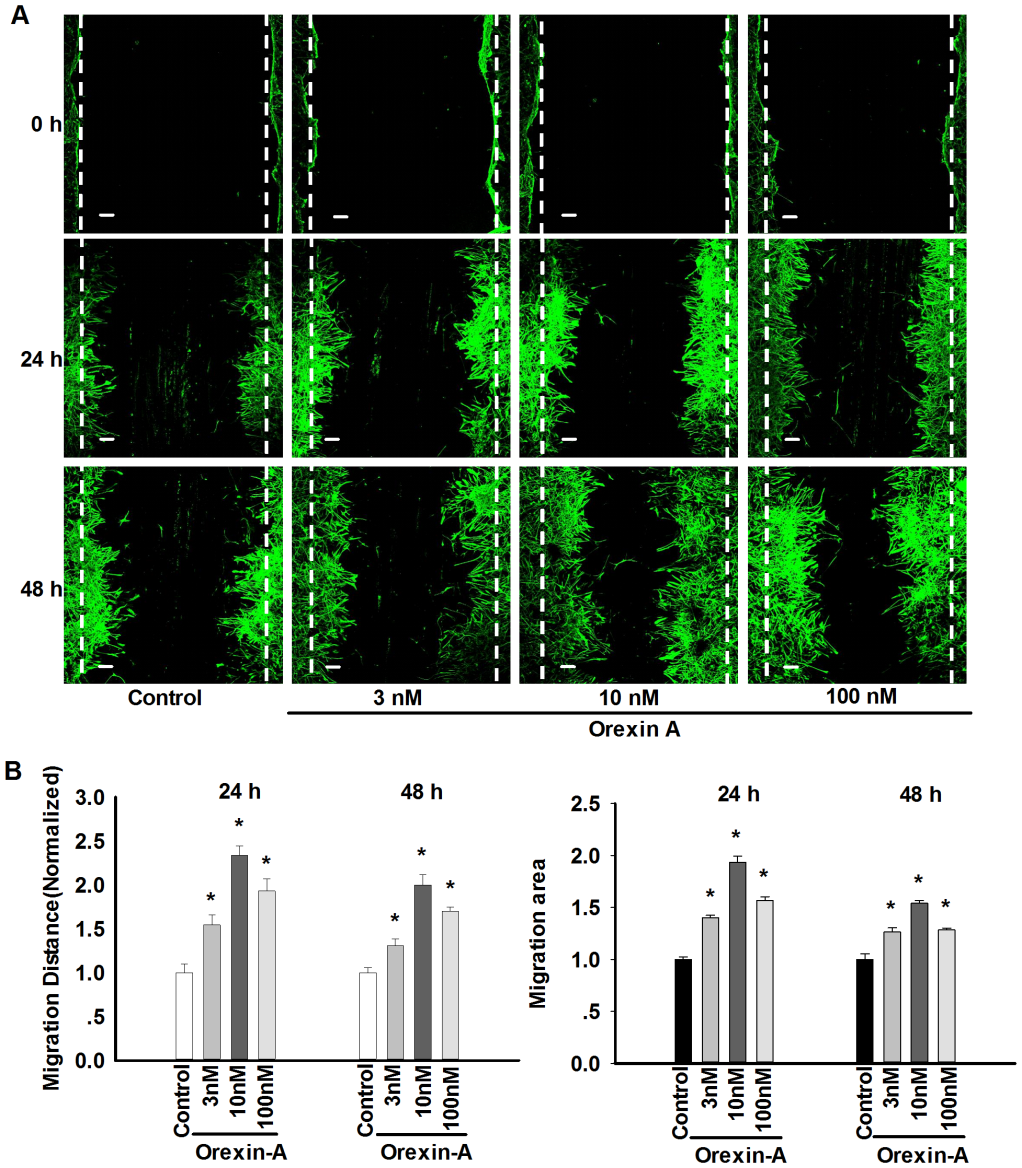
Orexin-A promotes cell migration in cultured rat astrocytes. A: The immunostaining photos indicating the promotion of astrocytes migration induced by orexin-A in different time and concentrations. The confluent monolayer of astrocytes was grown in serum deprived medium for 24 h, then scratched and 3, 10 and 100 nM orexin-A were added, photos were obtained at 0, 24 and 48 h. Astrocytes were immunostained with anti-GFAP. Scale bar  = 50 µm. B: Statistical analysis showing the promotion of astrocytes migration induced by orexin-A in different time and concentrations. Data are expressed as means ± SEM. n = 4, *p<0.05 *vs.* control.

To investigate the effect of orexin-A on cell migration in astrocytes, the confluent monolayer of astrocytes was grown in serum deprived medium for 24 h, then dishes were scratched, added with different concentrations of orexin-A (3 nM, 10 nM and 100 nM), then immunostained with anti-GFAP at 0, 24 and 48 h, respectively. It was found that orexin-A promoted astrocytes migration in different time and concentrations. It had a maximal effect at 10 nM and 24 h treatment (increased the migrating distance of the astrocytes into ∼240%, increased the migrating area of the astrocytes into ∼190%) (Fig. 1A–B), suggesting that orexin-A could promote the migration of cultured rat astrocytes.

### OX1R mediates orexin-A-induced cell migration in cultured rat astrocytes

**Figure 2:**
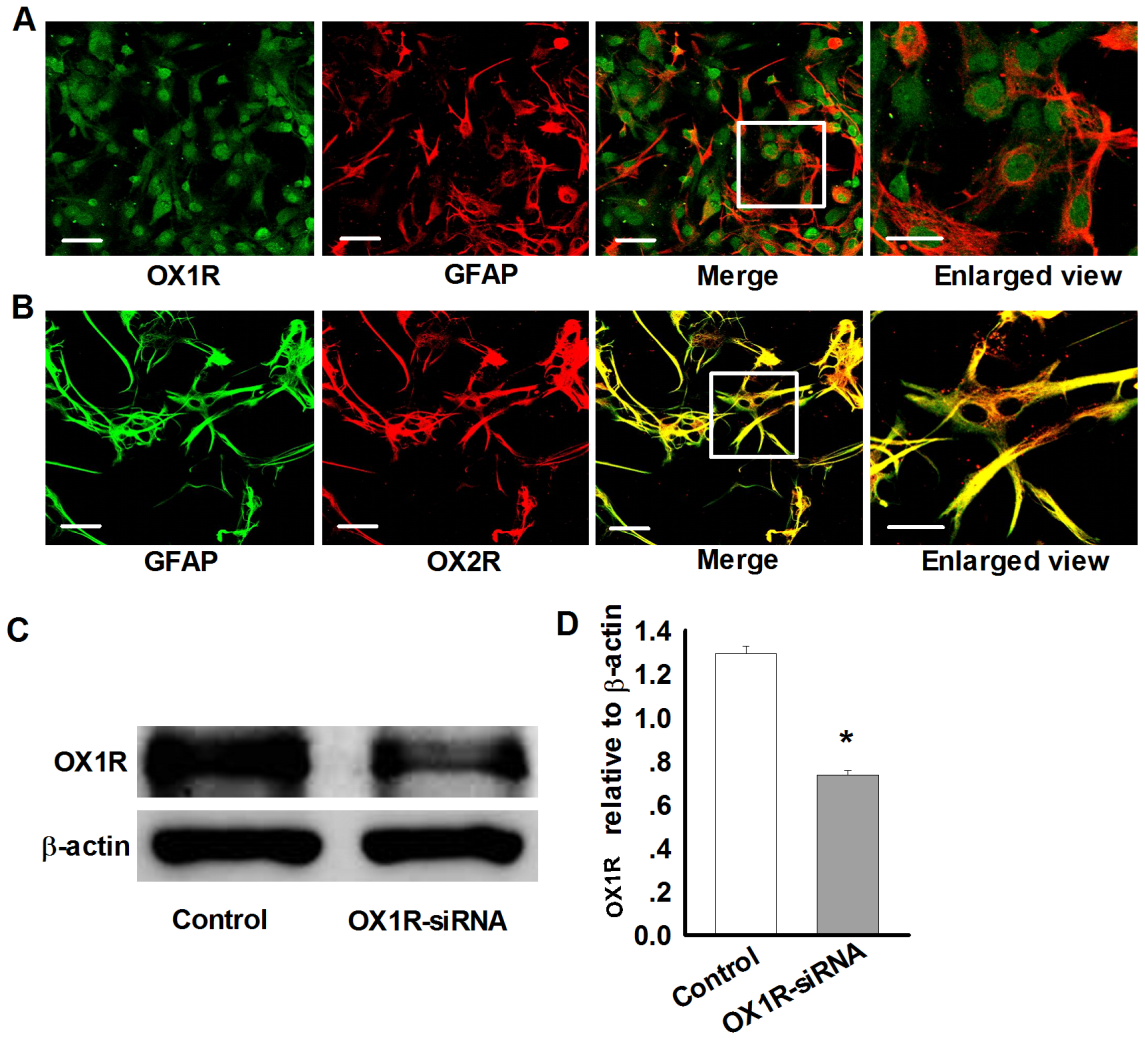
Both OX1R and OX2R are expressed in primary rat astrocytes. A: Colocalization of OX1R (Green) and GFAP (Red) in cultured rat astrocytes. n = 4, Scale bar  = 50 µm. The white pane indicating enlarged area. B: Colocalization of OX2R (Red) and GFAP (Green) in cultured rat astrocytes. n = 4, Scale bar  = 50 µm. The white pane indicating enlarged area. C–D: Representative western blots and statistical analysis showing the effect of siRNA-mediated silencing of OX1R expression in astrocytes. Data are expressed as means ± SEM. n = 3, *p<0.05 *vs.* control.

Previous study have reported that orexin-A promotes cAMP synthesis in astrocytes via OX1R [9], but they do not demonstrate whether orexin receptors express in astrocytes. Thus, by using anti-GFAP to label astrocytes, anti-OX1R and anti-OX2R to label orexin receptors, the results showed that both OX1R and OX2R expressed in rat astrocytes, but differently, OX1R expressed in plasma membrane, cytoplasm and nucleus, while OX2R only expressed in plasma membrane and cytoplasm (Fig. 2A–B). To further confirm our results, we used the methods of gene silencing. After adding the virus carrying the silent sequences, a significant reduction of OX1R expression was observed (Fig. 2C–D).

**Figure 3:**
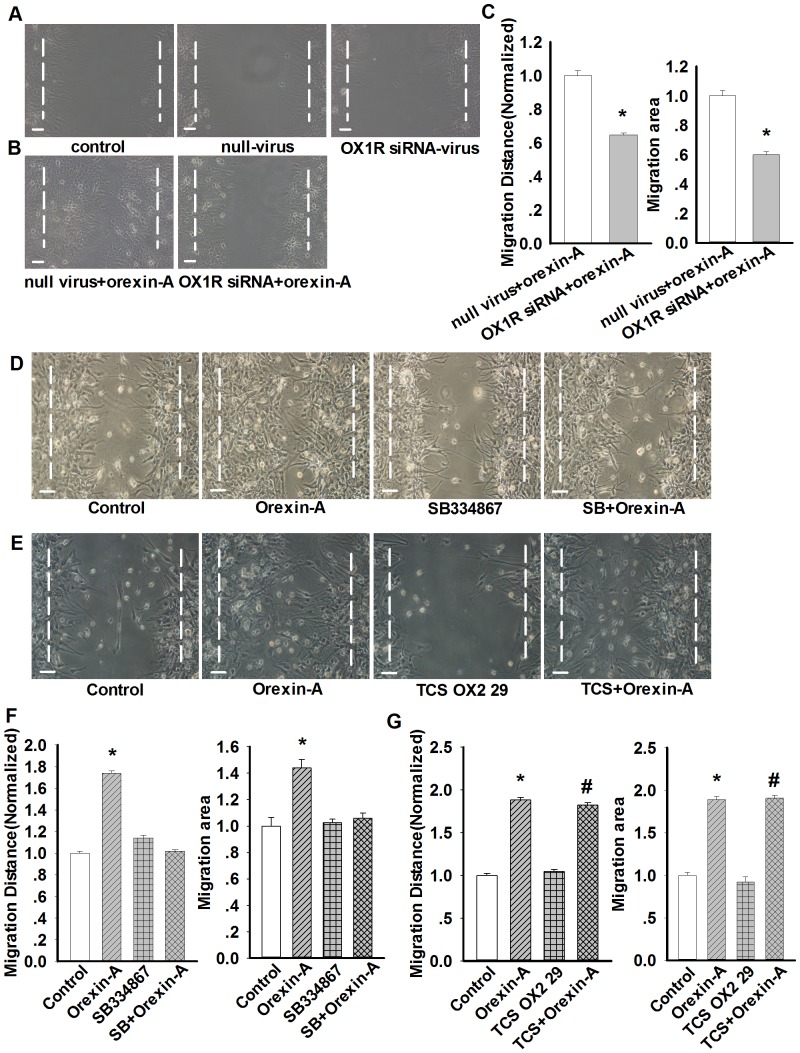
OX1R mediates orexin-A-induced astrocytes migration. A: Effects of viral vector on astrocytes migration. n = 4, Scale bar  = 20 µm. B–C: Representative wound healing images and statistical analysis showing the effects of siRNA-mediated silencing of OX1R on orexin-A induced astrocytes migration. Data are expressed as means ± SEM. n = 4, *p<0.05 *vs.* null virus+orexin-A. Scale bar  = 20 µm. D: Effects of specific OX1R inhibitor SB334867 (10 µM, 24 h) on orexin-A-induced astrocytes migration. n = 4, Scale bar  = 20 µm. E: Effects of specific OX2R inhibitor TCS OX2 29 (10 µM, 24 h) on orexin-A-induced astrocytes migration. n = 4, Scale bar  = 20 µm. F–G: Statistical analysis showing the effects of OX1R and OX2R inhibitors on orexin-A-induced astrocytes migration. Data are expressed as means ± SEM. n = 4, *p<0.05 *vs.* control, ^#^p<0.05 *vs.* TCS OX2 29.

We then determined whether these receptors mediate orexin-A-induced increase in astrocytes migration. Firstly, OX1R siRNA was used to silence OX1R gene, the results showed that viral vector (null or carrying OX1R siRNA) had no effects on primary astrocytes migration, however, silencing OX1R gene significantly inhibited orexin-A induced cell migration (Fig. 3A–C). Next, we used the antagonists of orexin receptors. Prior to orexin-A(10 nM) incubation, we pretreated astrocytes with a selective OX1R antagonist SB334867 (10 µM, 30 min). It was shown that SB334867 (10 µM, 30 min) pretreatment attenuated orexin-A-induced migration of astrocytes to control levels (Fig. 3D, 3F). However, pretreatment of OX2R antagonist TCS OX2 29 (10 µM, 30 min) had no effects on orexin-A-induced astrocytes migration (Fig. 3E, 3G). These results suggested that the function of orexin-A on astrocytes was mediated by OX1R.

### ERK1/2 signal mediated orexin-A-induced astrocytes migration

**Figure 4:**
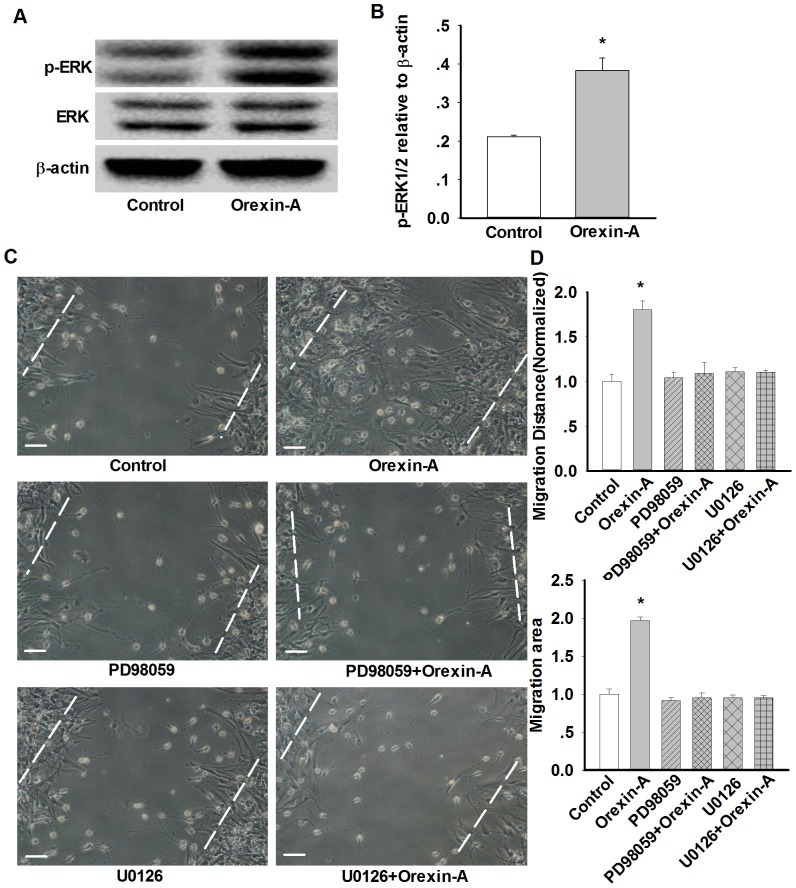
ERK1/2 signal mediates orexin-A-induced astrocytes migration. A–B: Representative western blots and statistical analysis showing the effect of orexin-A on p-ERK expression in astrocytes. Data are expressed as means ± SEM. n = 3, *p<0.05 vs. control. C–D: Representative wound healing images and statistical analysis showing the effect of ERK1/2 inhibitor PD98059 (20 µM, 24 h) and U0126 (10 µM, 24 h) on orexin-A-induced astrocytes migration. Data are expressed as means ± SEM. n = 4, *p<0.05 *vs.* control. Scale bar  = 20 µm.

To examine the relationship between orexin-A and ERK1/2 phosphorylation, primary cultured astrocytes were treated with orexin-A (10 nM) or vehicle for 10 min and the levels of ERK1/2 phosphorylation were assayed by western blot. As shown in Fig. 4A, orexin-A-induced phosphorylation of ERK1/2 was significantly elevated (0.38±0.03 in orexin-A 10 nM group *vs.* 0.21±0.01 in control group, n = 3, p<0.05) in primary cortical astrocytes, suggesting the possible contribution of ERK1/2 pathway in orexin-A-induced migration of astrocytes. To test this hypothesis, ERK1/2 inhibitors U0126 (10 µM, 30 min) and PD98059 (20 µM, 30 min) were added before orexin-A incubation. The migration result showed that orexin-A-induced astrocytes migration was prevented by U0126 and PD98059 significantly (Fig. 4C–D).

### Orexin-A-induced ERK1/2 phosphorylation and astrocytes migration are dependent on Ca^2+^ signaling pathway

**Figure 5:**
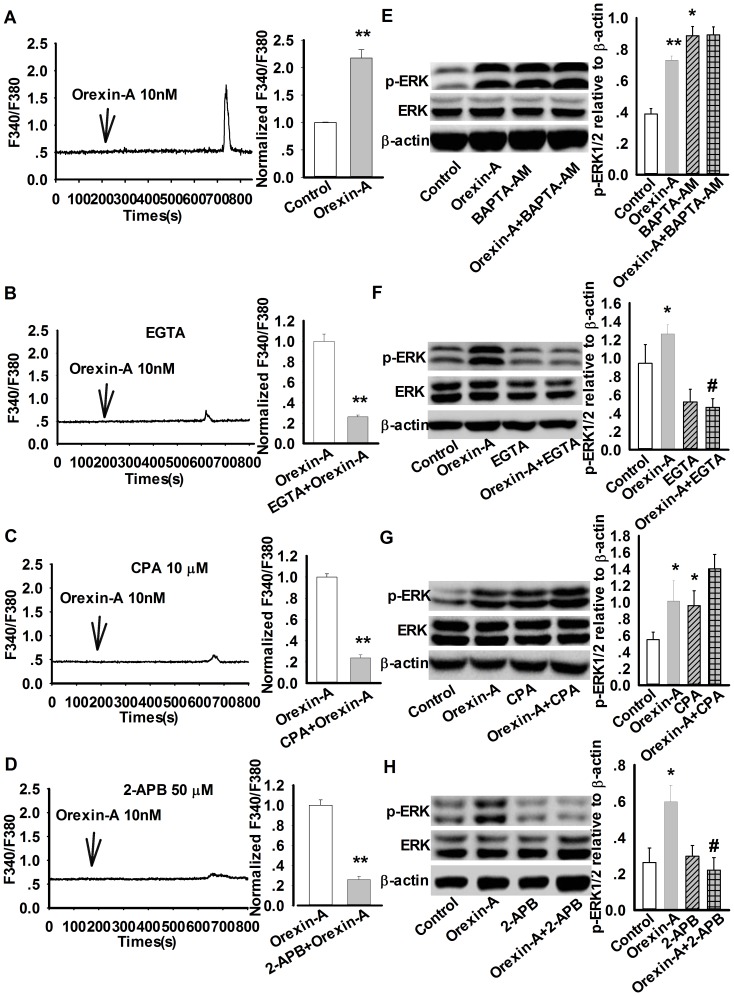
Orexin-A-induced ERK1/2 phosphorylation is dependent on Ca^2+^ signaling pathway. A: Calcium imaging result and statistical analysis showing the effect of orexin-A (10 nM) on intracellular Ca^2+^ concentration in astrocyte. Data are expressed as means ± SEM (n = 15, *p<0.05 *vs.* control). B–D: EGTA containing Ca^2+^-free solution, SR Ca^2+^-ATPase inhibitor CPA, and IP3Rs, SOC channel inhibitor 2-APB attenuated orexin-A-induced intracellular Ca^2+^ elevation. Data are expressed as means ± SEM. (B: EGTA, n = 17, **p<0.01 *vs.* orexin-A; C: CPA 10 µM, n = 15, **p<0.01 *vs.* orexin-A; D: 2-APB 50 µM, n = 15, **p<0.01 *vs.* orexin-A). E: Representative western blots and statistical analysis showing the effect of Ca^2+^ chelator BAPTA-AM (20 µM, 30 min) on orexin-A-mediated upregulation of p-ERK in astrocytes. Data are expressed as means ± SEM. n = 4, *p<0.05, **p<0.01 *vs.* control. F: Representative western blots and statistical analysis showing the effect of EGTA containing Ca^2+^-free solution on orexin-A-induced ERK1/2 phosphorylation. n = 3, *p<0.05 vs. control ^#^p<0.05 *vs* orexin-A. G: Representative western blots and statistical analysis showing the effect of SR Ca^2+^-ATPase inhibitor CPA (10 µM, 30 min) on orexin-A-induced ERK1/2 phosphorylation. n = 3, *p<0.05 vs control. H: Representative western blots and statistical analysis showing the effect of SOC inhibitor 2-APB (50 µM, 30 min) on orexin-A-induced ERK1/2 phosphorylation. Data are expressed as means ± SEM. n = 3, *p<0.05 *vs.* control, ^#^p<0.05 *vs* orexin-A.

Previous studies show that the binding of orexin-A with its receptor triggers an increase in intracellular calcium in some cell lines, which is coupled to ERK1/2 activation [16]. We therefore investigated whether intracellular Ca^2+^ participated in orexin-A-induced increase in ERK1/2 phosphorylation and subsequent cell migration in cultured astrocytes. Firstly, the changes in intracellular Ca^2+^ concentration by orexin-A were tested by calcium imaging. It was found that intracellular Ca^2+^ concentration increased notably about 500 sec after orexin-A (10 nM) application from 0.64±0.07 to 1.21±0.29 in fluorescence intensity (n = 15, p<0.01 Fig. 5A). The increases in intracellular Ca^2+^ can be derived from two sources: one is the mobilization of intracellular Ca^2+^ stores; the other is the influx of extracellular Ca^2+^ through membrane ion channels and ion transporters. So here, we firstly used Ca^2+^-free solution to evaluate the effects of extracellular Ca^2+^ on orexin-A-induced Ca^2+^ increase. It was found that orexin-A-induced Ca^2+^ increase was inhibited ∼70% in Ca^2+^-free solution (Fig. 5B), indicating that extracellular Ca^2+^ influx is necessary for orexin-A-induced intracellular Ca^2+^ elevation in astrocytes. As we known, the elevation of cytoplasmic calcium in non-excitable cells are mostly through the activation of inositol triphosphate receptors (InsP3Rs) and store-operated calcium (SOC) channels [17]. Therefore, we next applied 10 µM CPA (the inhibitor of endoplasmic reticulum Ca^2+^-ATPase) and 50 µM 2-APB (the inhibitor of InsP3Rs and SOC channel) 30 min before orexin-A administration respectively. The calcium imaging results showed that the orexin-A-induced Ca^2+^ elevation was inhibited significantly (CPA ∼75%, 2-APB ∼70%, Fig. 5C–D). These results illustrate that store-operated Ca^2+^ entry also contributed to the Ca^2+^ elevation induced by orexin-A in astrocytes.

In order to further explore the pathway of calcium signal, ERK1/2 phosphorylation was detected by western blot. Astrocytes were pretreated with a Ca^2+^ chelator BAPTA-AM (20 µM, 30 min), followed by application of orexin-A (10 nM) for 10 min. As shown in Fig. 5E, in spite of BAPTA-AM elevated the basal level of p-ERK (0.39±0.01 in control group *vs.* 0.86±0.05 in BAPTA-AM group, n = 4, p<0.05), it still prevented ERK1/2 phosphorylated continuously by orexin-A (0.88±0.05 in BAPTA-AM+orexin-A group *vs*. 0.86±0.05 in BAPTA-AM group, n = 4, p>0.05), suggesting that the increase of intracellular Ca^2+^ may contribute to orexin-A induced ERK1/2 phosphorylation. In order to confirm the role of intracellular Ca^2+^ in ERK1/2 activation, astrocytes were pretreated with different inhibitors. Firstly, Ca^2+^-free solution containing EGTA (the chelator of Ca^2+^) were administrated for 30 min before orexin-A treatment, we found that pretreatment of astrocytes with EGTA containing Ca^2+^-free solution significantly inhibited the increase in ERK1/2 phosphorylation in astrocytes (control: 1.01±0.23, orexin-A: 1.36±0.15, EGTA+orexin-A: 0.46±0.10, n = 3, p<0.05, Fig. 5E), indicating that extracellular Ca^2+^ entry contributes to the elevation of ERK1/2 phosphorylation. CPA (10 µM), the inhibitor of endoplasmic reticulum Ca^2+^-ATPase, was also employed. Similar with BAPTA-AM, CPA also elevated the basal level of p-ERK (0.55±0.09 in control group *vs.* 0.96±0.18 in CPA group, n = 3, p<0.05), but differently, it could not inhibit the activating effect of orexin-A on ERK1/2, the p-ERK1/2 level still raised from 0.96±0.18 to 1.40±0.17 (n = 3, p<0.05 *vs.* CPA group) (Fig. 5G), indicating that intracellular Ca^2+^ release may not contribute to orexin-A-induced ERK1/2 phosphorylation. Hence, we further examined whether store-operated Ca^2+^ entry (SOCE) mediated these effects. It was shown that 2-APB (50 µM, 30 min), a SOCE inhibitor, prevented the increase of p-ERK expression by orexin-A from 0.59±0.08 (orexin-A group) to 0.22±0.07 (2-APB+orexin-A group) (n = 3, p<0.05, Fig. 5H), indicating that SOCE mediates orexin-A-induced astrocytes Ca^2+^ influx and up-regulation of p-ERK protein.

**Figure 6:**
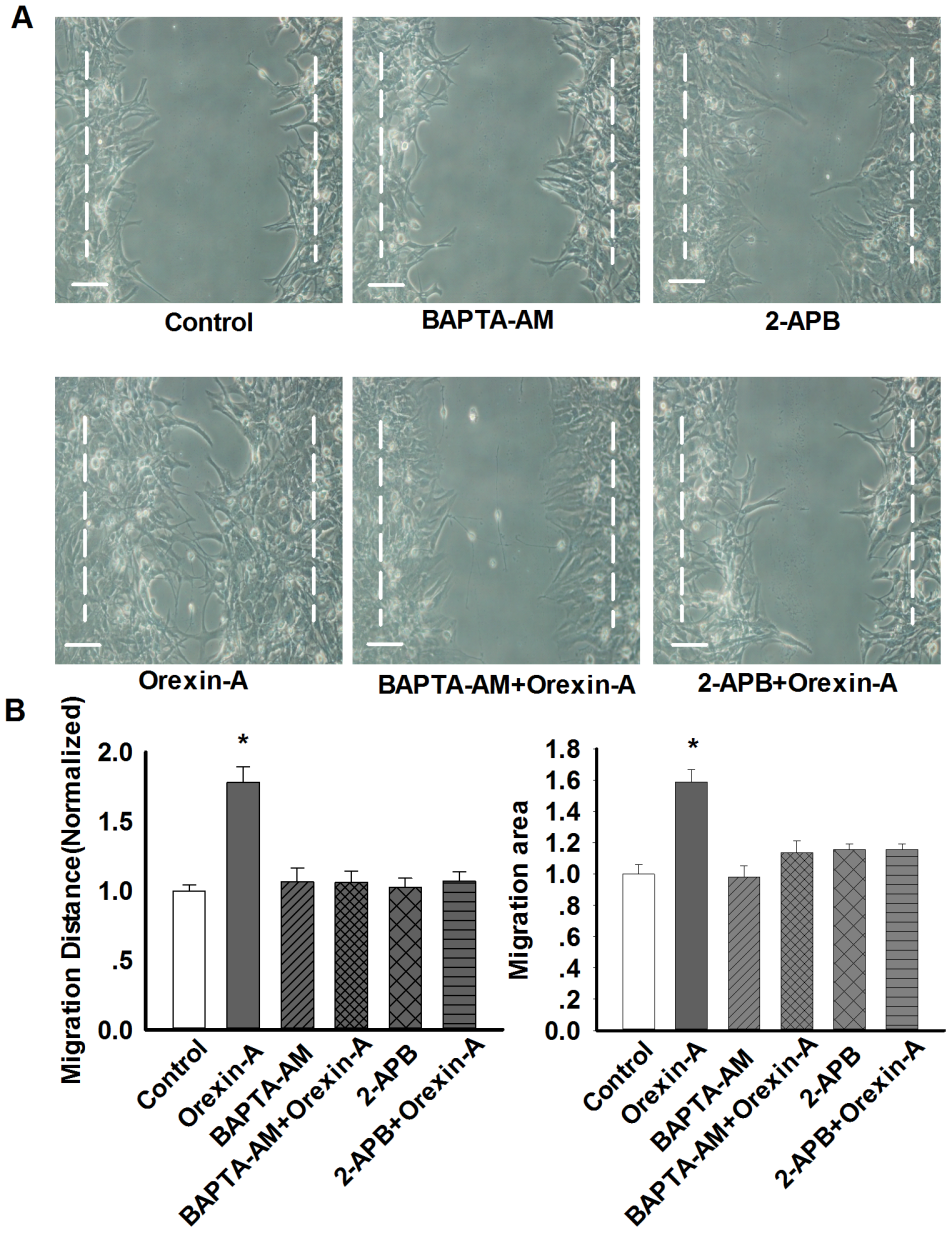
Orexin-A-induced astrocytes migration is dependent on Ca^2+^ signaling pathway. A: Representative wound healing images showing the effects of various Ca^2+^ pathway inhibitors on orexin-A-induced astrocytes migration. B: statistical analysis of wound healing assay. Data are expressed as means ± SEM, n = 5, *p<0.05 *vs.* control. Scale bar  = 20 µm. The images were selected from five individual experiments.

Finally, pharmacological inhibitors of calcium origins BAPTA-AM, 2-APB were used to examine the effects of calcium signal on cell migration with wound healing assay. As shown in Fig. 6, both BAPTA-AM and 2-APB completely prevented orexin-A-induced astrocytes migration. Above results suggest that SOCE- pathway-mediated extracellular Ca^2+^ influx was required for orexin-A-induced astrocytes migration.

### PLC signal is involved in orexin-A-induced astrocytes migration

**Figure 7:**
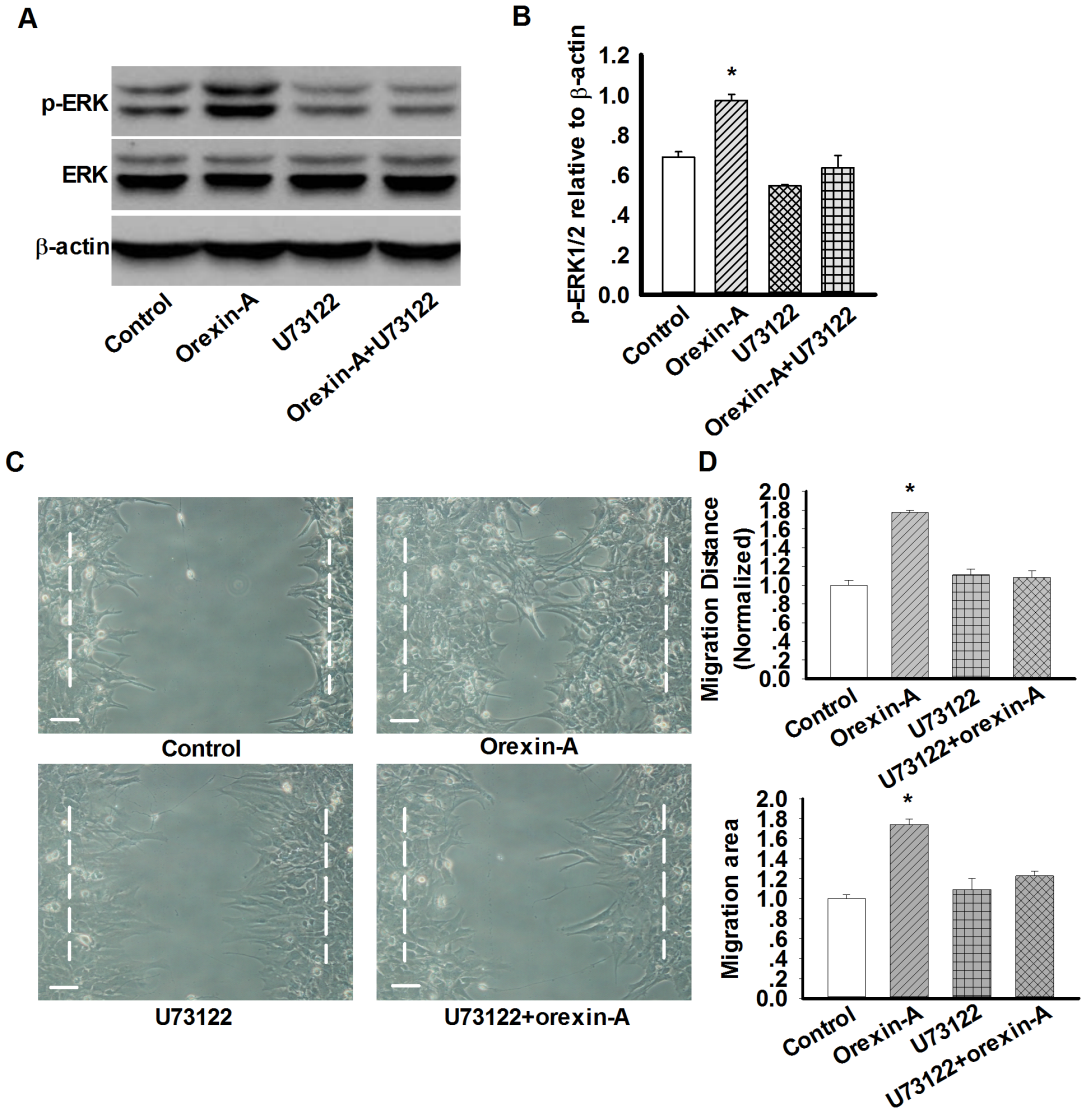
PLC-Ca^2+^ pathway mediate orexin-A-induced astrocyte migration. A–B: Representative western blots and statistical analysis showing the effect of PLC inhibitor U73122 (30 µM, 30 min) on orexin-A-induced ERK1/2 phosphorylation. n = 3, *p<0.05 *vs.* control. C–D: Representative wound healing images and statistical analysis showing the effect of PLC inhibitor U73122 on orexin-A-induced astrocytes migration. Data are expressed as means ± SEM. n = 3, *p<0.05 *vs.* control.

Since both BAPTA-AM and 2-APB inhibited ERK1/2 phosphorylation and astrocytes migration induced by orexin-A, we tested how PLC, an upstream molecule for mobilization of intracellular Ca^2+^, would affect orexin-A-induced ERK1/2 phosphorylation and migration. As expected, pre-incubation of 30 µM U73122, a specific inhibitor of PLC for 30 min, attenuated orexin-A (10 nM, 10 min)-evoked ERK1/2 phosphorylation (0.97±0.03 in orexin-A group *vs.* 0.67±0.03 in U73122+orexin-A group, n = 3, p<0.05 Fig. 7A-B), these results indicate that PLC signal participated in orexin-A-induced ERK1/2 phosphorylation. Next, U73122 (30 µM, 30 min) was used in cell migration test, the result displayed that U73122 completely prevented orexin-A-induced astrocytes migration to control level (Fig. 7C–D), indicating that PLC signal was involved in orexin-A-induced astrocytes migration.

### PKCα is necessary for orexin-A-induced increase in ERK1/2 phosphorylation and cell migration of cultured rat astrocytes

**Figure 8:**
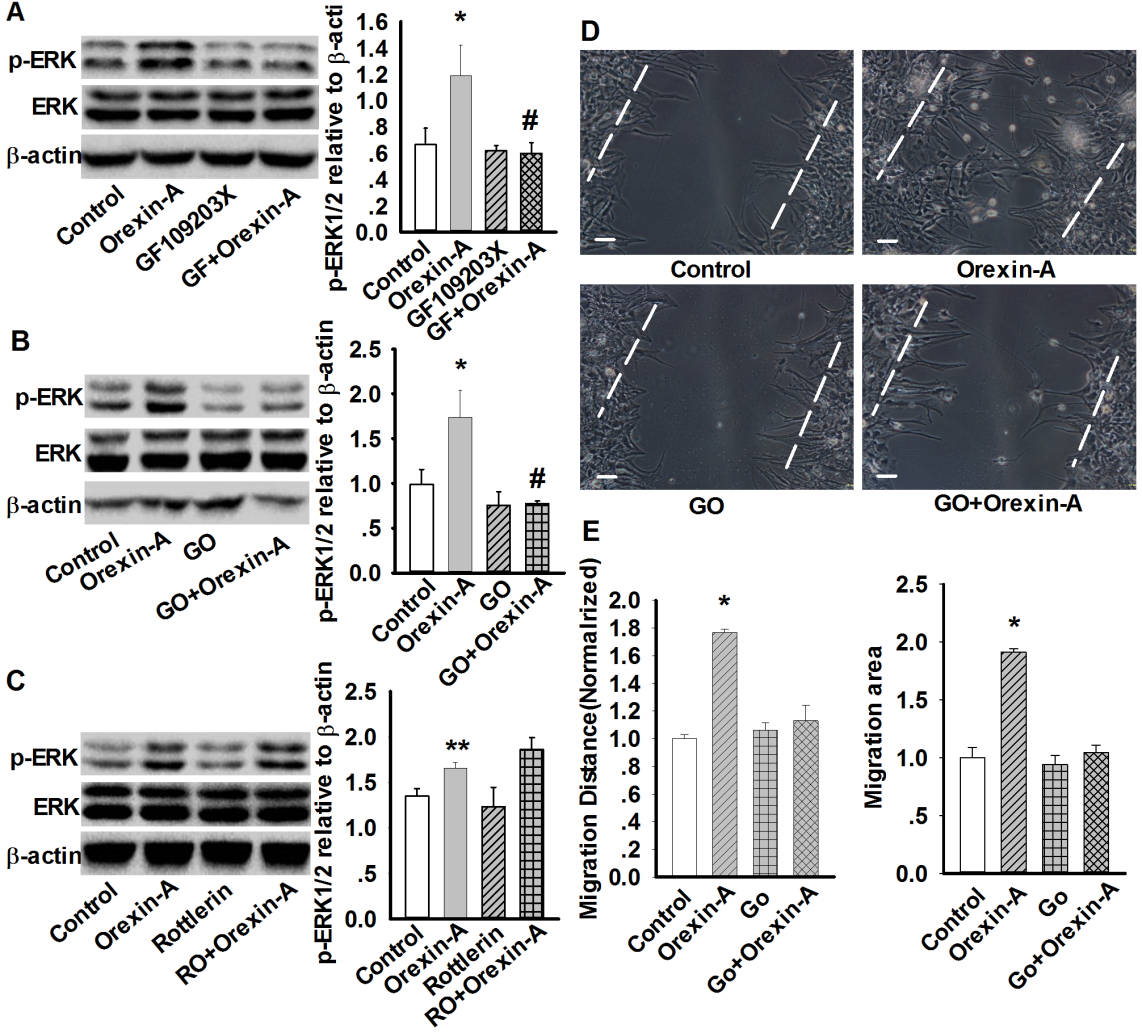
PKCα mediates orexin-A-induced upregulation of p-ERK and cell migration in astrocytes. A: Effect of PKC inhibitor GF109203X (10 µM, 30 min) on orexin-A-induced ERK1/2 phosphorylation. Data are expressed as means ± SEM. n = 3, *p<0.05 *vs.* control, ^#^P<0.05 *vs.* orexin-A (GF: GF109203X). B: Effect of PKCα inhibitor Gö6976 (1 µM, 30 min) on orexin-A-induced ERK1/2 phosphorylation. Data are expressed as means ± SEM. n = 3, *p<0.05 *vs.* control, ^#^p<0.05 *vs.* orexin-A (GO: Gö6976). C: Effect of PKCδ inhibitor rottlerin (5 µM, 30 min) on orexin-A-induced ERK1/2 phosphorylation. Data are expressed as means ± SEM. n = 3, **p<0.01 *vs.* control (RO: Rottlerin). D: Representative wound healing images showing PKCα inhibitor (GO: Gö6976) prevented orexin-A-induced astrocytes migration. E: Statistical analysis of wound healing assay. Data are expressed as means ± SEM, n = 5, *p<0.05 *vs.* control. Scale bar  = 20 µm. The images were selected from five individual experiments.

PKC is one of the molecules downstream of PLC activation. We then determined whether orexin-A-induced ERK1/2 phosphorylation attributed to PKC activation. As shown in Fig. 8A, non-selective PKC inhibitor GF109203X (10 µM, 30 min) significantly inhibited the increase in ERK1/2 phosphorylation induced by orexin-A from 1.19±0.22 to 0.52±0.01 (n = 3, p<0.05) (Fig. 8A), suggesting that PKC is indeed involved in orexin-A-induced ERK1/2 phosphorylation.

PKC has various isoforms that possess different characteristics. For example, activation of the typical PKCα isoform is dependent on both Ca^2+^ and DAG, but activation of the novel PKCδ isoform is dependent on DAG but not on Ca^2+^ [18]. We therefore investigated the roles of different PKC isoforms in orexin-A-induced ERK1/2 phosphorylation using various pharmacological inhibitors. Gö6976 (1 µM, 30 min), a specific inhibitor of PKCα, significantly reduced the orexin-A-induced increase in ERK1/2 phosphorylation from 1.74±0.31 to 0.77±0.02 (n = 3, p<0.05 Fig. 8B), while the PKCδ inhibitor rottlerin (5 µM, 30 min) could not prevent the effect of orexin-A induced ERK1/2 phosphorylation in astrocytes (1.65±0.06 in orexin-A group *vs.* 1.86±0.13 in rottlerin+orexin-A group, n = 3) (Fig. 8C). In line with the effect of Gö6976 (1 µM, 30 min) on orexin-A-induced ERK1/2 phosphorylation, Gö6976 markedly attenuated orexin-A-induced astrocyte migration to control level (Fig. 8D–E). These results indicate that PKCα functionally mediates the increase in ERK1/2 phosphorylation and cell migration induced by orexin-A in cultured astrocytes.

## Discussion

Orexin-A, a multifunctional neuropeptide in the nervous systems, is originally discovered in the hypothalamus in 1998 [19]. The peptide exerts its diverse effects via activation of OX1R or OX2R. Neuronal OX1R activation participates in many physiological and pathophysiological processes such as wake/sleep regulation [20], energy homeostasis modulation [21], and depression pathological mechanisms [22]. However, the functions of orexin-A in astrocytes are largely unknown. In this study, we examined the functional responses and mechanisms of astrocytes to orexin-A, and found that orexin-A enhanced astrocyte migration via OX1R-PLC-Ca^2+^-PKCα signaling pathway. Notably, astrocytes migration is important in brain inflammation and remodeling during brain injuries such as brain wound healing, tissue remodeling, and glial scar formation [23]–[25]. Based on our results, orexin-A may participated in the brain inflammation and neural remodeling.

Astrocytes are involved in various pathological conditions through their membrane receptors such as the classical cannabinoid-like receptors [26], dopamine receptors [18] and AMPA receptors [8]. Orexin-A can activate both OX1R and OX2R, but no studies have reported their existence in astrocytes. Here, we demonstrated the expression of OX1R and OX2R proteins in astrocyte in different manner. OX1R was present in the cytoplasm and the nucleus, while OX2R was found only in the cytoplasm. One previous study shows that only specific OX1R antagonist SB334867 can prevent orexin-A-induced cAMP synthesis in astrocytes, and OX2R antagonist TCS OX2 29 has no effect [9]. This result indicates that OX1R and OX2R mediate different functions in astrocytes. Thus, we next investigated which receptor mediated orexin-A-induced astrocytes migration. Similarly, only specific OX1R inhibitor SB334867 prevented orexin-A- induced astrocytes migration, OX2R inhibitor TCS OX2 29 had no effects. These results strongly suggested that the effect of orexin-A on astrocyte is mainly mediated by OX1R.

Recent studies have reported that orexins can activate ERK1/2 associated signals, which plays crucial roles in the CNS [27]. Besides, orexin-A activates ERK1/2 signal in CHO and vascular endothelial cell [13], [28], but no reports in astrocyte. Since ERK1/2 is important to cell proliferation and migration [29], we hypothesized that orexin-A increased astrocyte activity (e.g. migration) via promoting ERK1/2 activation. Here, we found that orexin-A induced ERK1/2 phosphorylation in cultured rat astrocytes. ERK1/2 inhibitors U0126 and PD98059 can strongly prevent orexin-A induced astrocytes migration. These results suggest that ERK1/2 signal participates in the effect of orexin-A on astrocytes migration. However, further experiments are needed to investigate the role of other signaling pathways in astrocyte migration induced by orexin-A.

In endothelial cells and CHO cells, OX1R activation causes a robust increase in intracellular Ca^2+^ level [13], [28]. Here, we also observed a similar phenomena, a Ca^2+^ spike was appeared about 500 sec after orexin-A treatment in astrocytes. The results indicate that intracellular Ca^2+^ elevation may be involved in orexin-A-induced ERK1/2 phosphorylation and astrocyte migration. Such a long delay between adding orexin-A and the Ca^2+^ spike might due to indirect method of administration. However, when we used Ca^2+^ chelator BAPTA-AM to explore the detail mechanisms, we found that the basal level of ERK1/2 phosphorylation increased by BAPTA-AM, which was in accordance with our previous study [18]. The increased ERK1/2 phosphorylation may be explained as inhibition of intracellular Ca^2+^ increase and elimination of negative Ca^2+^-calcineurin signals by BAPTA-AM [31], since Ca^2+^ generally binds to calcineurin, which physically interacts with ERK1/2 and restrains its activity through de-phosphorylation [30]. However, ERK1/2 phosphorylation was not elevated continuously when co-administration BAPTA-AM with orexin-A, suggesting that intracellular Ca^2+^ signal participated in orexin-A-induced ERK1/2 activation. In addition, some calcium origin blockers had the similar inhibitory effects. Intracellular Ca^2+^ increase was mainly derived from two sources: the Ca^2+^ store mobilization [32], [33], such as endoplasmic reticulum (ER) and SOC [34], and influx of extracellular Ca^2+^. After administration of EGTA (a chelator of extracellular Ca^2+^) containing Ca^2+^-free solution, orexin-A-induced increase in intracellular Ca^2+^ and ERK1/2 phosphorylation was considerably inhibited, suggesting that extracellular Ca^2+^ was necessary for orexin-A-induced ERK1/2 phosphorylation. The similar results were obtained by 2-APB (SOC inhibitor) administration. However, CPA (inhibitor of the endoplasmic reticulum Ca^2+^-ATPase) could not arrest orexin-A-induced astrocytes ERK1/2 phosphorylation. Taken together, these results showed that SOC-mediated extracellular Ca^2+^ influx was necessary for orexin-A-induced ERK1/2 phosphorylation and migration in astrocytes.

OXRs are known to induce intracellular Ca^2+^ elevation via both receptor-operated Ca^2+^ channels (ROCs) and PLC-Ca^2+^ release via SOCs [34]. PLC is an enzyme responsible for the hydrolysis of the membrane phospholipid phosphatidylinositol-4, 5-bisphosphate (PIP2) to inositol-1, 4, 5-trisphosphate (IP3) and diacylglycerol [35]. IP3 activates IP3 receptors leading to the release of Ca^2+^ from the ER and additional influx of extracellular Ca^2+^ through SOCs [36]. Here, we found that pretreatment of astrocytes with U73122, a specific PLC inhibitor and 2-APB, the inhibitor of both IP3 receptors and SOCE, markedly suppressed orexin-A-induced increase in ERK1/2 phosphorylation and following cell migration. Thus, PLC-Ca^2+^ pathway via SOCs extracellular Ca^2+^ influx was needed for orexin-A-induced astrocytes migration.

PKC is an upstream kinase for ERK1/2 activation [37]. In addition, the PLC/PKC pathway is important for ERK1/2 activation. Firstly, we found that non-selective PKC inhibitor GF109203X could notably prevented Orexin-A-induced ERK1/2 phosphorylation. As we known, the PKC family can be divided into three subfamilies: conventional (or classical) PKCs containing isoforms of α, βI,βII, and γ, novel PKCs (δ, ε, η, and θ), and atypical PKCs (ζ and λ) [18]. Every PKC subunit has different characteristics, for example, the conventional PKCs are Ca^2+^-dependent and activated by DAG, while, the novel PKCs are Ca^2+^-independent and also activated by DAG, but the atypical PKCs are both Ca^2+^ and DAG-independent [18]. Thus, we further clarified the detailed isoforms that are involved in orexin-A-induced ERK1/2 phosphorylation and astrocytes migration. We investigated the respective roles of the different PKC isoforms on ERK1/2 phosphorylation. It was found that PKCα inhibition robustly attenuated orexin-A-induced ERK1/2 phosphorylation and astrocyte migration, whereas PKCδ was not involved in ERK1/2 phosphorylation. This result potentially provided us a new target to explore the functions of orexin-A.

In conclusion, our results clearly demonstrated that OX1R-PLC-Ca^2+^-PKC pathway mediates the orexin-A-induced ERK1/2 activation and then contributes to migration in cultured rat astrocytes. It is demonstrated for the first time that orexin-A can regulate cell migration, which might help us to understand the new physiological and pathophysiological roles of orexin-A in the central nervous system.
