# Correction: Orexin-A Promotes Cell Migration in Cultured Rat Astrocytes via Ca^2+^-Dependent PKCα and ERK1/2 Signals

**DOI:** 10.1371/journal.pone.0251224

**Published:** 2023-10-11

**Authors:** Qing Shu, Zhuang-Li Hu, Chao Huang, Xiao-Wei Yu, Hua Fan, Jing-Wen Yang, Peng Fang, Lan Ni, Jian-Guo Chen, Fang Wang

In Fig 8A, the representative β-actin blot is incorrect. Please see the correct Fig 8 here.

**Fig 8:**
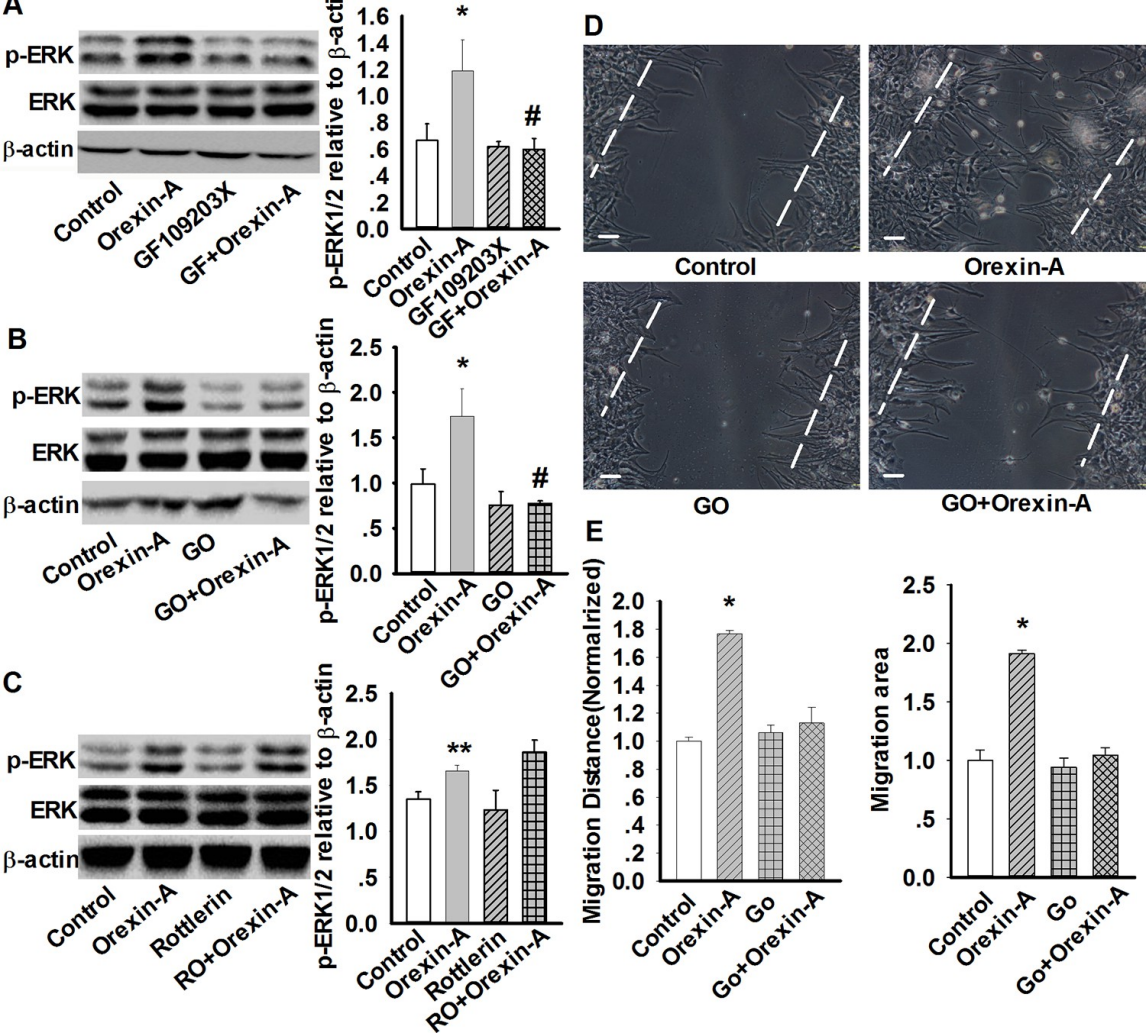
PKCα mediates orexin-A-induced upregulation of p-ERK and cell migration in astrocytes. A: Effect of PKC inhibitor GF109203X (10 μM, 30 min) on orexin-A-induced ERK1/2 phosphorylation. Data are expressed as means ± SEM. n=3, *p<0.05 vs. control, #P<0.05 vs. orexin-A (GF: GF109203X). B: Effect of PKCα inhibitor Gö6976 (1 μM, 30 min) on orexin-A-induced ERK1/2 phosphorylation. Data are expressed as means ± SEM. n=3, *p<0.05 vs. control, #p<0.05 vs. orexin-A (GO: Gö6976). C: Effect of PKCδ inhibitor rottlerin (5 μM, 30 min) on orexin-A-induced ERK1/2 phosphorylation. Data are expressed as means ± SEM. n=3, **p<0.01 vs. control (RO: Rottlerin). D: Representative wound healing images showing PKCα inhibitor (GO: Gö6976) prevented orexin-A-induced astrocytes migration. E: Statistical analysis of wound healing assay. Data are expressed as means ± SEM, n=5, *p<0.05 vs. control. Scale bar =20 μm. The images were selected from five individual experiments.

S1 File and S2 File are not listed as Supporting Information. They can be viewed below.

## Supporting information

S1 File Fig 5G Raw Images. (PDF)

S2 File Fig 8A Raw Images. (PDF)
