# Supplementary figures and images for: Correction: Orexin-A Promotes Cell Migration in Cultured Rat Astrocytes via Ca2+-Dependent PKCα and ERK1/2 Signals
Source: PLoS One. 2023 Oct 11;18(10):e0251224. doi: 10.1371/journal.pone.0251224 (PMC10566686; doi:10.1371/journal.pone.0251224)

Uncropped western blots in Fig 5G (n=3)

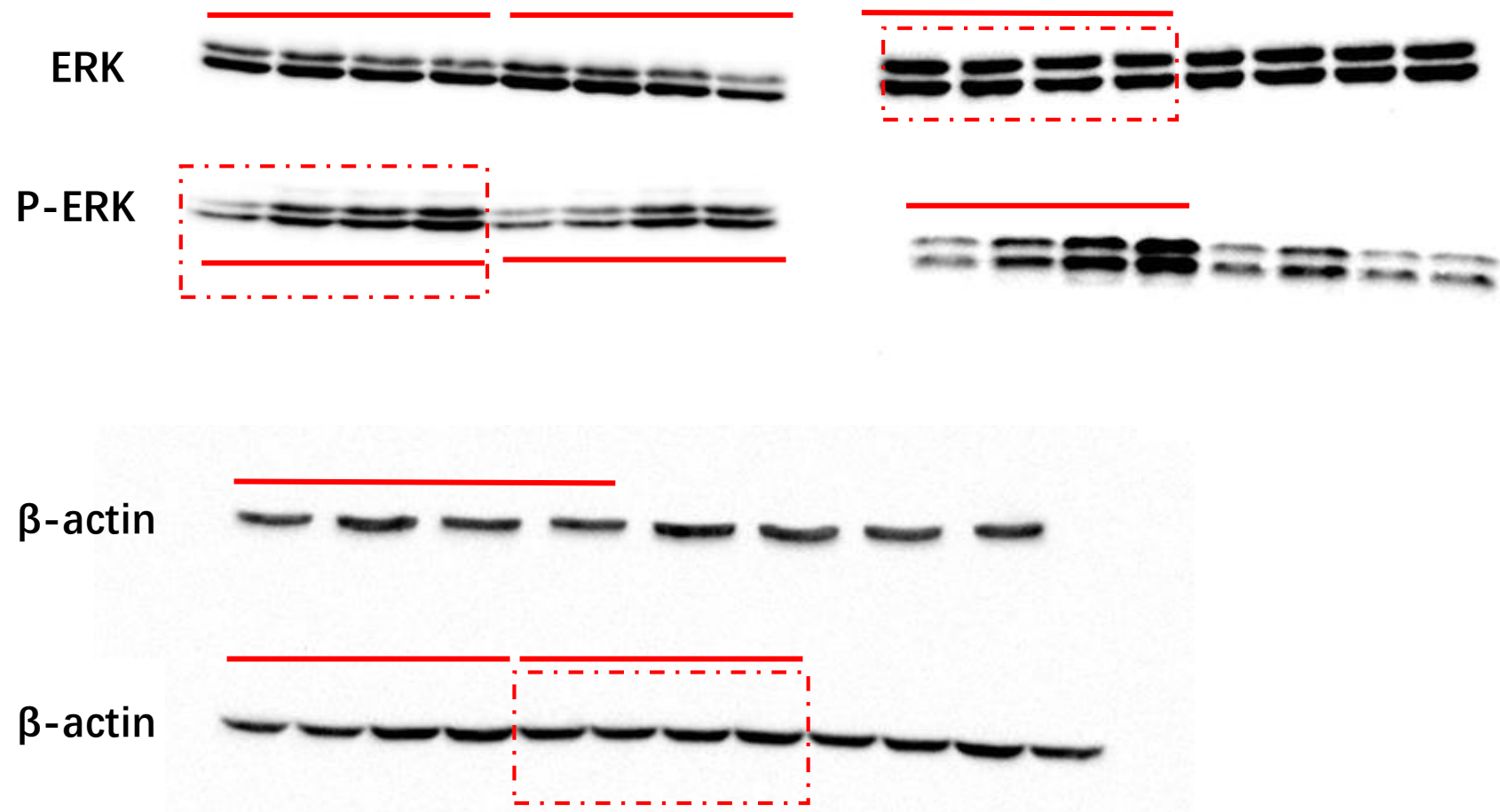

Supplement: S1 File — (PDF) [file pone.0251224.s001.pdf]

### Uncropped western blots in Fig 8A (n=3)

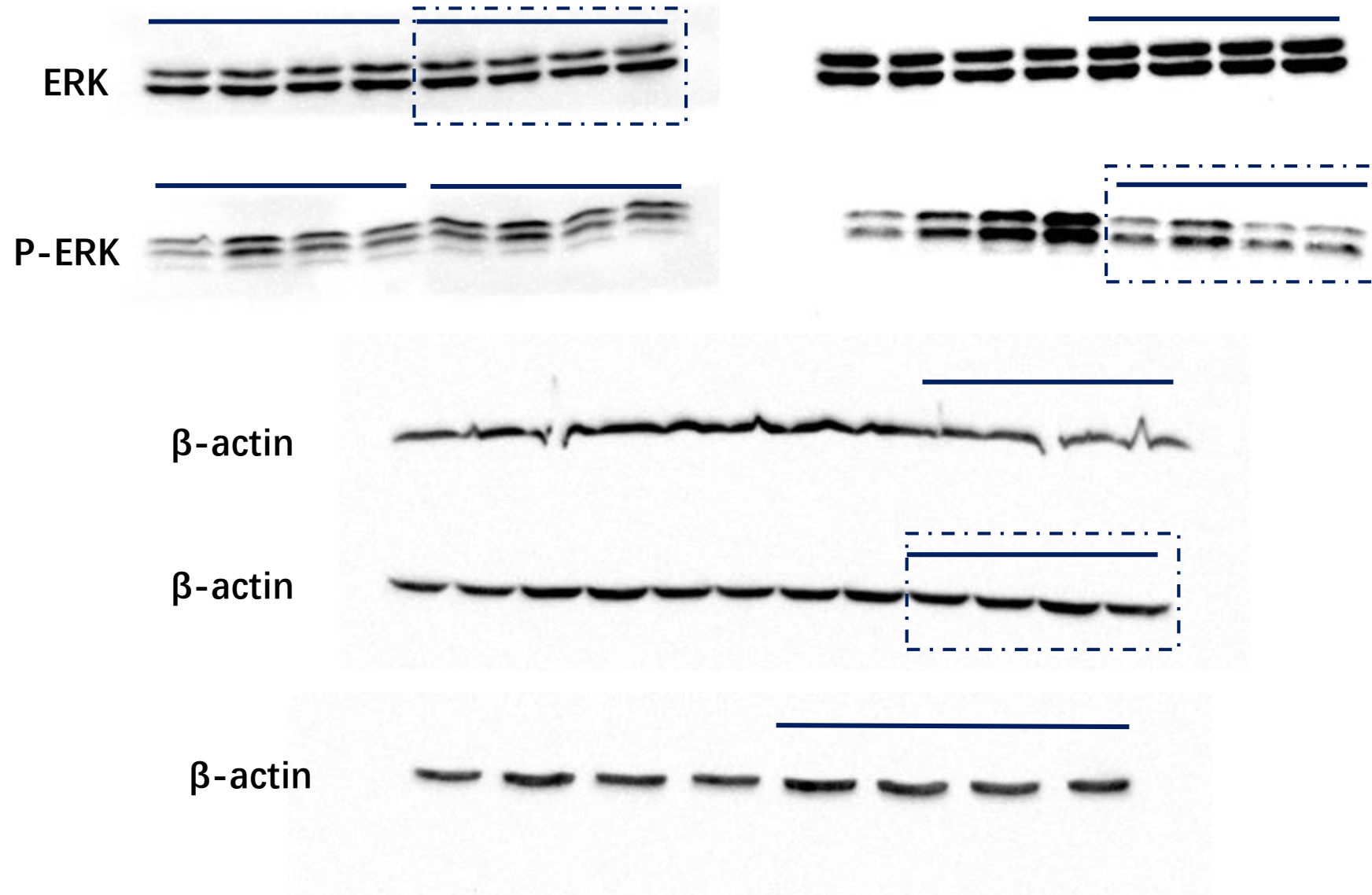

Supplement: S2 File — (PDF) [file pone.0251224.s002.pdf]
